# Using Depth Cameras to Detect Patterns in Oral Presentations: A Case Study Comparing Two Generations of Computer Engineering Students

**DOI:** 10.3390/s19163493

**Published:** 2019-08-09

**Authors:** Felipe Roque, Cristian Cechinel, Tiago O. Weber, Robson Lemos, Rodolfo Villarroel, Diego Miranda, Roberto Munoz

**Affiliations:** 1 Centro de Ciências, Tecnologias e Saúde, Universidade Federal de Santa Catarina, Araranguá 88906072, Brazil; 2 Escuela de Ingeniería Informática, Pontificia Universidad Católica de Valparaíso, Valparaíso 2362807, Chile; 3 Escuela de Ingeniería Civil Informática, Universidad de Valparaíso, Valparaíso 2362735, Chile

**Keywords:** MS Kinect, multimodal learning analytics, oral presentations, k-means, educational data mining

## Abstract

Speaking and presenting in public are critical skills for academic and professional development. These skills are demanded across society, and their development and evaluation are a challenge faced by higher education institutions. There are some challenges to evaluate objectively, as well as to generate valuable information to professors and appropriate feedback to students. In this paper, in order to understand and detect patterns in oral student presentations, we collected data from 222 Computer Engineering (CE) fresh students at three different times, over two different years (2017 and 2018). For each presentation, using a developed system and Microsoft Kinect, we have detected 12 features related to corporal postures and oral speaking. These features were used as input for the clustering and statistical analysis that allowed for identifying three different clusters in the presentations of both years, with stronger patterns in the presentations of the year 2017. A Wilcoxon rank-sum test allowed us to evaluate the evolution of the presentations attributes over each year and pointed out a convergence in terms of the reduction of the number of features statistically different between presentations given at the same course time. The results can further help to give students automatic feedback in terms of their postures and speech throughout the presentations and may serve as baseline information for future comparisons with presentations from students coming from different undergraduate courses.

## 1. Introduction

The development of communication and teamwork skills has been indicated by professional organizations [1] and curricula recommendations and by reports for university degree programs [2] as fundamental expertise for the preparation of future professionals, in different areas of knowledge. Regarding communication skills, the primary goal is to convey the information clearly and coherently to the people [3]. Good communicators demonstrate their presentation skills with verbal and non-verbal characteristics, such as body language, eye contact with the audience, or the space they occupy on stage [4]. Body language is an important part of the learning and communication processes [5]. Additionally, body postures and gestures are used in oral presentations to convey ideas and messages [6].

Today, advanced technologies and sensors are available in learning environments that allow capturing data through different modalities [7]. According to Ochoa [8], the field of Multimodal Learning Analytics (MMLA) attempts to incorporate different sources of learning traces into Learning Analytics (LA) research, other than the traditional log-file, captured by online systems. Advanced sensors technologies allow for capturing biometric data with different modalities. This includes artifacts, such as gaze [9], postures [5,10], actions [11], facial expressions [12], speech [7,13], writing [14], and sketching [15]. These artifacts are examples of sources multimodal information that have been gradually incorporated into systems of learning.

There are some challenges associated with the combination of data from various sources, resulting in noisy results [16]. Problems with data from a variety of sources and programs, involving facial and speech recognition, usually have complex resolutions, which are often be solved through machine learning techniques [17,18]. Furthermore, the identification and characterization of undergraduate students is a fundamental starting point, in order to obtain better education and learning processes [19]. In the evaluation of students using MMLA techniques, after capturing data from a group of users and different sources, the data sets should be merged for statistical processing or data mining, to extract reasonable information from it. Machine learning techniques, which create models from past data, are important in areas where there are large data analyses. The MMLA, along with machine learning techniques, is a powerful approach assisting in the development of education [20], especially in the analysis of student behavior during oral presentations.

This work presents an approach for the identification of patterns in student presentations using multimodal data from the speech and body information that the students have collected through Microsoft Kinect. The approach consists of the application and evaluation of clustering techniques (i.e., silhouettes and K-means), in order to find the distinct categories and profiles of those presenters. For this, groups of undergraduate students from two different years (2017 and 2018) have been considered, based on the experimental studies for oral presentations during the project course of the academic year. The final objective is the development of a system capable of automatically recognizing the profiles of the presenting students during an oral presentation, in order to allow immediate feedback on the performed presentations. For the present study, the following research questions are proposed:

- RQ1: How many and which are the different groups of patterns found in the students’ oral presentations?
- RQ2: Which are the similarities and differences among the presentations of the different years?
- RQ3: Is it possible to observe patterns in the learning outcomes of students from both years in terms of corporal postures?

The paper continues as follows. Section 2 discusses some relevant works related to MMLA and postural patterns. Section 3 presents material and methods as well as the case study, namely two groups of Computer Engineering Students from different generations performing oral presentations for a course project. Section 4 presents and discusses the key results of our experiments. Finally, Section 5 ends the paper with conclusions and propositions for future work.

## 2. Related Work

Multimodal Learning Analytics is a field related to LA with emphasis on the analysis of complex communication modalities during learning activities such as speech, writing and nonverbal interaction [21]. Although relatively young, MMLA is an active field and a variety of techniques and applications have been proposed in the last few years [22].

A usual approach in MMLA is the integration of camera recordings with other data, in order to analyze student behavior. For instance, Bidwell et al. [23] used a multi-camera video recording and observers’ classifications to propose a behavior analysis framework. Their goal was to model and classify student engagement from sequences of student gaze targets, using face tracking software. Additionally, a group of expert observers classified the student behaviors into categories (engaged, attentive or transition). With both of these inputs, a student engagement classifier was then created to provide a report to the teacher. In addition, the study presented in [24] video, audio and pen stroke information, which were used to discriminate between experts and non-experts in groups of students solving mathematical problems.

Another instance of multi-camera video recordings being used in classroom contexts is the work of Raca and Dillenbourg [25], in which three to four cameras were used to collect data in a regular classroom, in an unobtrusive way. Then, gaze and body motion information were extracted from the videos to provide a report to the professor. In [26], in addition to the collected images, questionnaires, interviews and an eye-tracker were also used in the experiment.

In the more specific context of postural patterns, many previous works have used traditional cameras to collect data [27,28,29,30,31]. Using a Kinect device, a study [32] proposed an alternative to solve problems that would otherwise be difficult to address using conventional cameras in the recognition of human postures. They used the skeletal tracking tool provided by the Kinect SDK to collect joint information. Based on the tracked skeleton, joint positions and angles were used as features. These were then used in the support vector machine (SVM) technique to recognize human postures. Munoz et al. [5] developed a tool to capture, classify (using AdaBoost algorithm) and visualize the body postures of students, using a Kinect device, also allowing the use of data from other sensors.

Echeverria et al. [33] also used the Microsoft Kinect device to detect common postures from the body joints of the skeleton. They then employed the fuzzy C-Means to extract generalizations of postures, per person, and then performed clusterization using the K-Means algorithm.

Relly, Ravanell and Schneider analysed Kinect and speech data collected during a robot programming task [34]. They showed the correlation between certain movements and gesture patterns with learning gains. They also used clustering algorithms to find recurrent body position categories, which were then used to analyze the amount of time students spent on them and learning gains.

MMLA has also been used to provide oral presentation feedback to teachers and students. Presentations are an important way for humans to convey information to large groups. As a consequence, students are often evaluated with respect to these presentations. One approach to oral presentation feedback is to use only low-cost sensors, to provide data from audio and video [10] or from audio and slides [35]. Kinect devices can also be used to collect data for presentation feedback. In the previously mentioned work of Echeverria et al. [33], a set of predictors based on video and Kinect data were used to estimate the presentation skills of students. From the video records, they estimated the presenter’s gaze and other descriptive values. From the Kinect records, the authors extracted common postures and features from body joints of the skeleton information.

In the work by Gan et al. [36], a more intensive use of sensors required traditional cameras, Kinect records and Google Glass sensors to create a multi-sensor presentation analytics framework.

## 3. Materials and Methods

**Figure 1:**
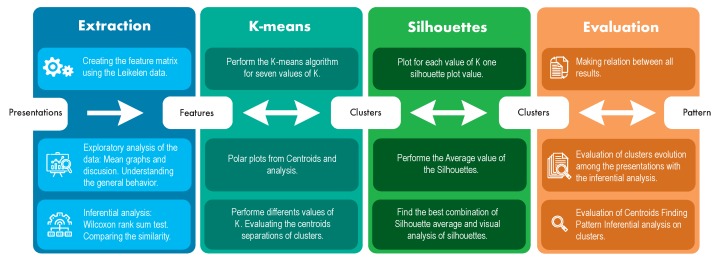
General scheme of the methodology.

This section presents the methodology adopted in the paper. The section also shows a description of the data collected and also the database used. Figure 1 shows a general outline of the methodology and procedures used to obtain the results.

### 3.1. Case Description

**Figure 2:**
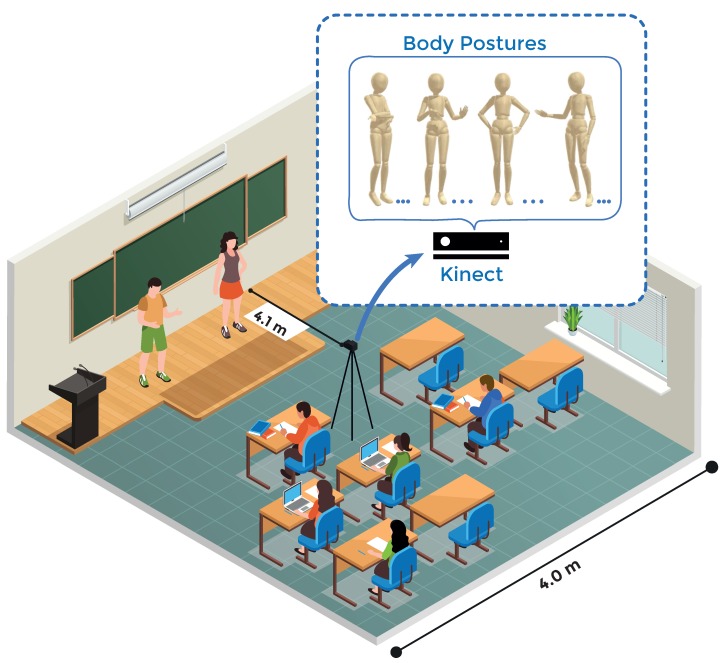
Setting environment.

The learning context of the present study is the “Introduction to Engineering” course offered to Computer Engineering (CE) students of the Universidad de Valparaíso, in Chile, during the years 2017 and 2018. During the course, students performed three oral group presentations about three topics previously predefined in the outline course as will be shown in Section 3.3. A scheme of the setting environment is presented in Figure 2.

### 3.2. Microsoft Kinect

The data capture for this work was done with Microsoft (MS) Kinect v2. MS Kinect is capable of capturing up to six people simultaneously and proved to be efficient in extracting the posture and speech patterns necessary for the experiments. Its advantages are related to the number of sensors present in the device. It contains a depth sensor, a color camera, and a full microphone array that provides full-body 3D motion capture, facial recognition, and voice recognition capabilities [37].

**Figure 3:**
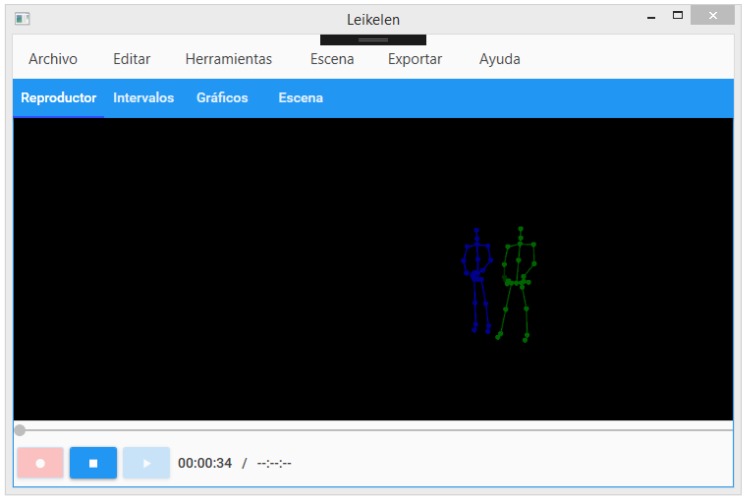
Lelikelen application screen showing the skeleton models of two people.

We used skeletal tracking to classify student positions and voice detection. With this, it was possible to represent the human body as a number of joints. These joints are body parts: arms, neck, hands, and head—for example, Figure 3 shows two people and their representations by skeletal tracking. These representations of skeletal tracking were used to classify pre-defined and trained actions and thus assemble the databases used. The time spent in each posture/action was stored as well.

### 3.3. Procedures and Data Collection

The data captured corresponds to oral presentations in the Introduction to Engineering course. The course is offered in the first semester to the students of the Informatics Engineering degree (at Universidad de Valparaíso, Chile). In the course, students must perform three evaluated oral presentations, associated with (1) web development, (2) microcontrollers, and (3) database (with one month between each presentation). Each presentation was performed by groups of two students, with a maximum of 5 min per group. PowerPoint slides could support all the student presentations. All presentations were captured using the MS Kinect v2 and Lelikelen software (https://github.com/leikelen-team/Leikelen). The students had to sign an informed consent at the beginning of the course. Moreover, the methodology was approved by the Research with Human Beings Ethics Committee of the Medicine Faculty at the Universidad de Valparaíso.

### 3.4. Lelikelen Tool

We have used the Lelikelen [5] software for data gathering and processing (see Figure 3). The software allows one to detect, store, and visualize body postures of the recorded people. Lelikelen has options to add custom postures, to export and import scenes, and to add a viewer of the postures detected, together with a timeline. Finally, Lelikelen also allows one to export the data to be visualized using other types of data mining tools.

Lelikelen uses Microsoft Kinect to capture body positions and the algorithm ADABoost [38] to classify the postures. The software has 10 predefined and validated body postures, together with other kinds of metrics such as (1) proxemic distance (distance between two or more people) [39], (2) whether the person is looking at the audience, (3) the inclination of the body and (4) whether the person is speaking. For this work, the postures used were incorporated from [5], being mainly based on [33,40].

#### 3.4.1. Features Collected

The features used in the present work are shown in Table 1. The raw data are composed of a sequence of periods and the respective student action/posture, captured by the system for that given period. For instance, a student can spend a certain period with his arms crossed, and then let his arms down for a very short period of time, and then start pointing to the slides, finally returning to the position with his arms crossed. For this sequence, we obtain four records composed of the actions/postures captured (CrossArms, HandsDown, Point, and CrossArms), together with the period that each action/posture lasted [5].

We collected a total of six different students data sets during the years of 2017 and 2018 (three presentations each year), as shown in Table 2.

#### 3.4.2. Data Description

Usually, an observation *E* is represented by a collection of attribute values (characteristics) (*x*_1_; *x*_2_; …; *x_n_*), where *x_i_* value of an attribute *X_i_*. For this problem, an example is a student in a presentation. The observations of this problem were obtained through the sum of the time intervals of each attribute, divided by the total time of presentation, thus the maximum possible for a given observation of an attribute is 1.

Table 3 shows the average values of each attribute in each dataset. The values are normalized (ranging from 0 to 1) and were obtained from the sum of the values of the given attribute in the database divided by the number of observations in the dataset:

(1) $$\bar{A}=\frac{\sum_{j=1}^{N}A_{j}}{N},$$

where $\bar{A}$ is the average value for an attribute, *N* is the number of observations given a presentation and *A_j_* is the value of an attribute in the database. As can be seen in Table 3, some attributes are more prevalent than others. The attribute that occurs the most in the data sets is the posture *Straight*. For the three presentations of 2017, this attribute occurred for more than 90% of the total time, while for the year 2018, the attribute occurred for around 80% of the total time. The *WatchingPublic* and *HandsDown* postures also occur quite frequently and stand out from the others. The higher occurrences for these first two attributes (*Straigth* and *WatchingPublic*) are expected, as during the presentation it is quite normal for the students to be standing and constantly looking at the public. The top 5 attributes that were prevalent in each presentation are presented in Table 4. For 2017, the top 5 attributes that occurred the most are almost the same, with a slight difference in the ranking (except for the attribute *OpenHands* in P3). The same goes for 2018, where the difference among the presentations is for the attribute *CrossArms* in P3.

As shown in Table 3, some attributes do not vary significantly across presentations. For instance, *CrossArms* and *HandsDown* present very similar values along the presentations of both years. On the other hand, some attributes vary across presentations and in comparison between the years. These differences are better analyzed in Section 4, where we compare the characteristics of the data sets and discuss the most important patterns found among them.

## 4. Results

### 4.1. Comparing the Attributes between Data Sets

**Figure 4:**
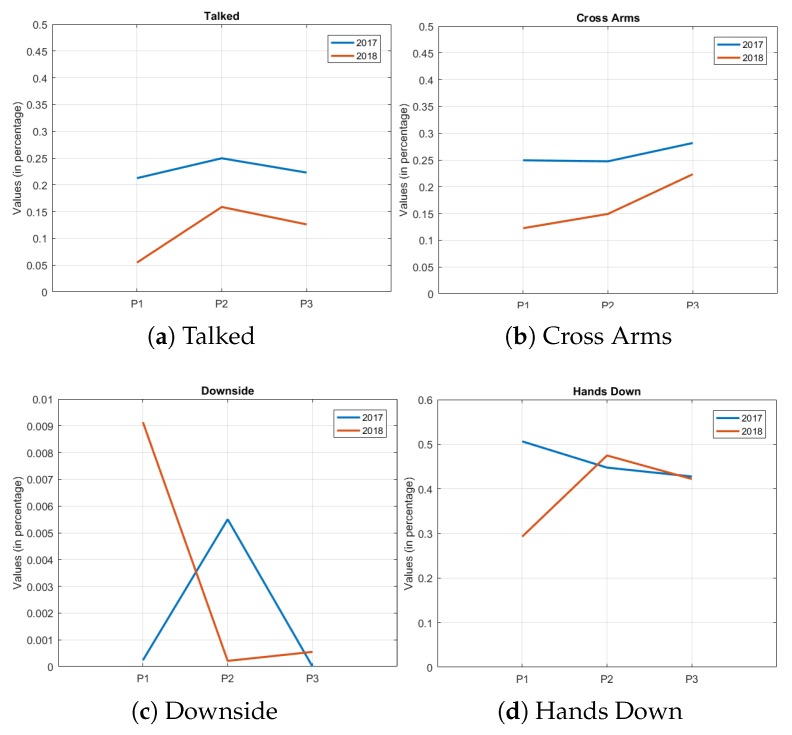
Trend graphs between the years.

One way of comparing the years evaluated in the study is to generate trend graphs between the years 2017 and 2018. When analyzing the graphs, it is possible to observe attributes with a similar trend (where the values of the attributes of one year follow the ones of the other), while others where this behavior is not observed and the graphs have random behaviors. However, another behavior, where, at some point (throughout the presentations), the values of the attributes are very close, can be seen in Hands on Face. These results are shown in Figure 4.

The first group of attributes (similar values of evolution throughout the presentations) have a very clear similarity and can be observed in the chart of talked and Cross Arms, for example, Figure 4a,b. In this group, the behavior is the same in the three presentations of the semester; if one value of the variable falls in one year in the second presentation, it also falls in the same presentation of the other year. It should be noted that this decrease or increase in attribute value varies between years and in intensity.

The other group of attributes are those of differences in behavior throughout the presentations, such as the downside, Figure 4c. The values of time percentages are different between years, and there is apparently no similarity of behavior between the evaluated years. What can be noticed is that the values are close between the years, even in this group of attributes where the trend that was shown previously is not observed. It is still possible to observe attributes where, at some point in the presentations, they obtained a similar trend, but that diverged in another point in the semester, as in the attribute Hands Down, Figure 4d.

In order to better compare the databases, some inferential statistics metrics were used. First, we analyzed the null hypothesis that the attributes come from a standard normal distribution [41]. The results showed that none of the attributes have normal distribution for those two years. Thus, it was necessary to use a non-parametric method to test the null hypothesis that data and two different databases are samples from continuous distributions with equal medians.

The Wilcoxon rank sum test [42] is a non-parametric test for two populations, when the samples are independent. The Wilcoxon rank sum test is equivalent to the Mann–Whitney U-test. For this work, the *p*-value was used to indicate the equality of the two population medians, at a 5% level significance. High values of *p* indicate that there is not enough evidence to reject the null hypothesis. Low values of *p* indicate otherwise.

Table 5 presents the tests for each pair of two presentations (databases), thus combining all possibilities, totaling 15 comparisons between databases. To facilitate the analysis, the comparisons between the presentations of different moments, of different years, were excluded. Consequently, the table shows only comparisons between the same years and comparisons between the same moments of different years. In order to make the reading of the table cleaner, the values considered small were rounded to 0. As can be seen, some combinations obtained low values of *p*. This indicates that, in these attributes, populations have statistically different medians. The values that are in bold represent the results that have statistically different medians.

In the year 2017, the medians obtained statistically similar values. In comparison of the first presentation with the second one, for example, only one attribute was statistically different. For the comparison between the first and third presentations, we had 0 attributes statistically different. However, in the last comparison, between the second and third presentations, two attributes tested as different. Still, for the year 2017, only *Downside* two tests did not present similarities among the medians.

Already for the presentations of 2018, more attributes presented significant differences. The first comparison, between presentations one and two, obtained three attributes considered statistically different; compared to 2017, this can be considered a similar result, since we had 12 attributes to evaluate. The comparison between the first and third presentations had one attribute with different medians. In the last comparison of 2018, between the second and third presentations, we had 0 tested statistically different medians. In 2018, *Downside* two tests again presented a statistically different median.

Comparing the two years with the presentations at the same moments (moments in sequence), it is possible that, during the presentations, the attributes differences were reduced from 6 to 1. In the comparison with the first presentation of the two years (2017 and 2018), there were more statistical differences of the attributes, with 6. The first presentations are those that contain more observations in the databases, which can generate some variation.

As mentioned, in the comparison of the first presentations, we have a total of six statistically different attributes. We can visualize that, in the comparison between the second presentations for each year, the value falls from 6 to 3, an indication that the students present (in relation to the evaluated attributes) more similarity between the years. Finally, in comparison with the last presentations, we see that the different attributes statistically fall to 1. A new fall in the total number of different attributes between the years, which can indicate that, in the end, the students presented in a more similar pattern with respect to the two years.

### 4.2. Clustering

In order to evaluate the context, where presentation patterns are found, some algorithms were used to explore the data. The K-means algorithm groups examples similar to the same set, according to their characteristics. In this way, the values of the centroids can be analyzed. Even so, in many cases, it is difficult, and several times it is not known how many groups the problem has. The *Silhouettes* algorithm aids in this sense, assisting visually in identifying cases where the K-means is misplacing some examples.

#### 4.2.1. K-Means

K-means [43] is an iterative data partitioning algorithm that assigns *i* observations to exactly one of the *k* clusters defined by centroids. The value of *k* is defined before the algorithm starts, and clustering is achieved by minimizing the sum of the distances of the squares between the data and the centroids of the clusters. In this work, the algorithm K-means was used to partition student data into *k* clusters.

#### 4.2.2. Silhouettes

To explore the data and analyze the groups created by K-means, the visual analysis algorithm Silhouette [44] was used. This method is useful for selecting the best number of predefined clusters. It can also be used to exchange an observation that has a negative Silhouette value for its neighbor, thus improving the analysis results of the [44] groups. The value of Silhouette is defined by the Equation (2), as follows:

(2) $$s\left(i\right)=\frac{b(i)-a(i)}{max[a(i),b(i)]},$$

where *a*(*i*) is the average distance between an example *i* and all other data within the same cluster. This implies that *a*(*i*) is how well the given *i* object is inside your cluster. *b*(*i*) is the shortest average distance of *i*, for all points in any other cluster, of which *i* is not part. Hence, the closer to 1 the value of *s*(*i*), so is the chance of belonging to the cluster assigned *i*. Similarly, negative values represent that this example does not belong to the cluster. Many values close to 0 are at the border between the clusters, and can therefore vary between the two.

#### 4.2.3. The Clusters

**Figure 5:**
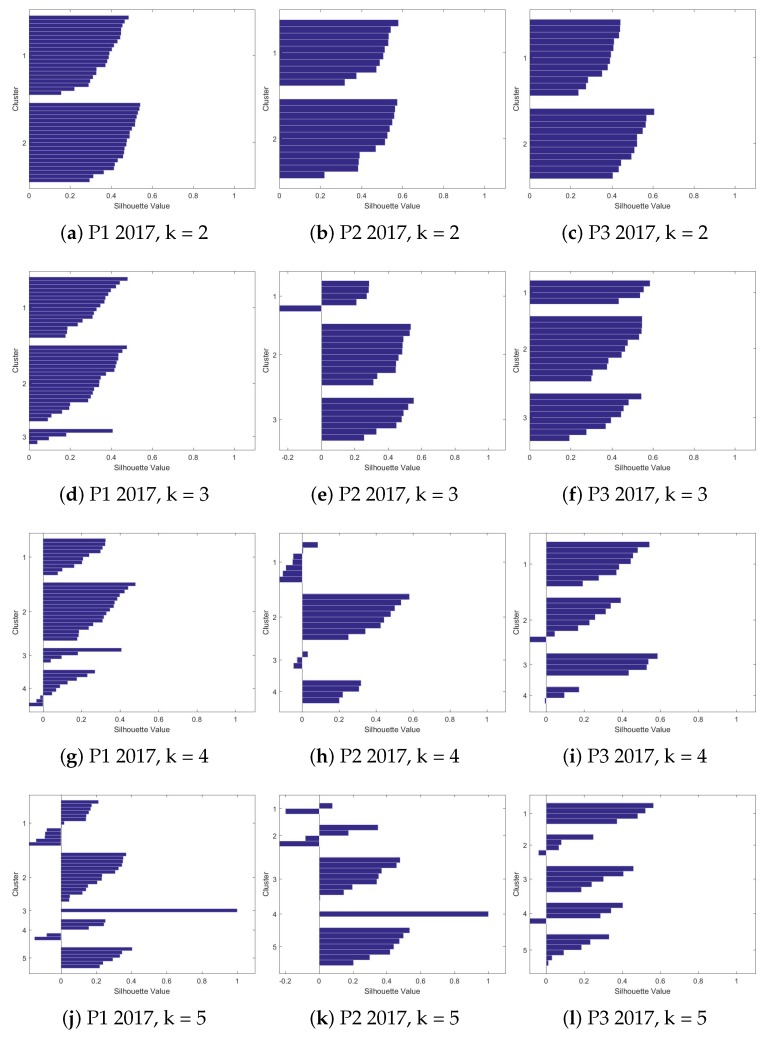

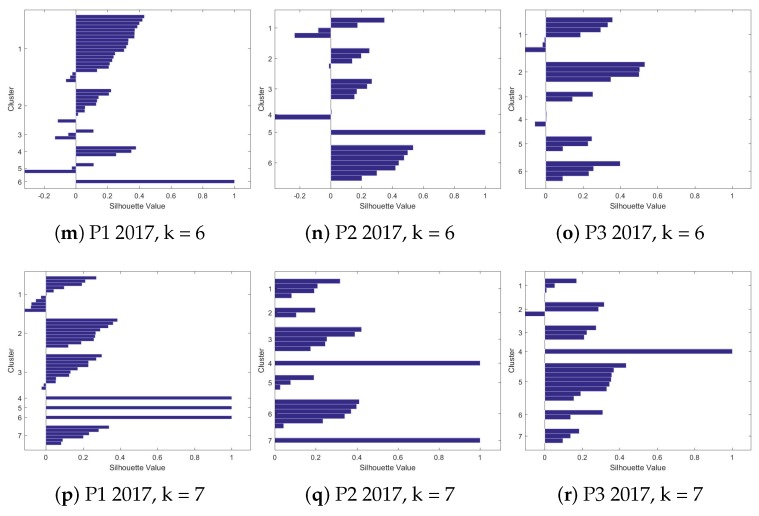
Silhouettes’ charts for three different times in 2017.

**Figure 6:**
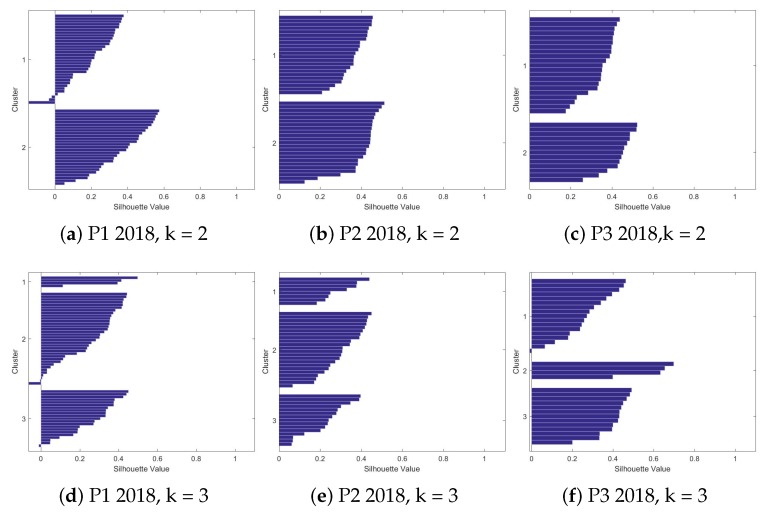

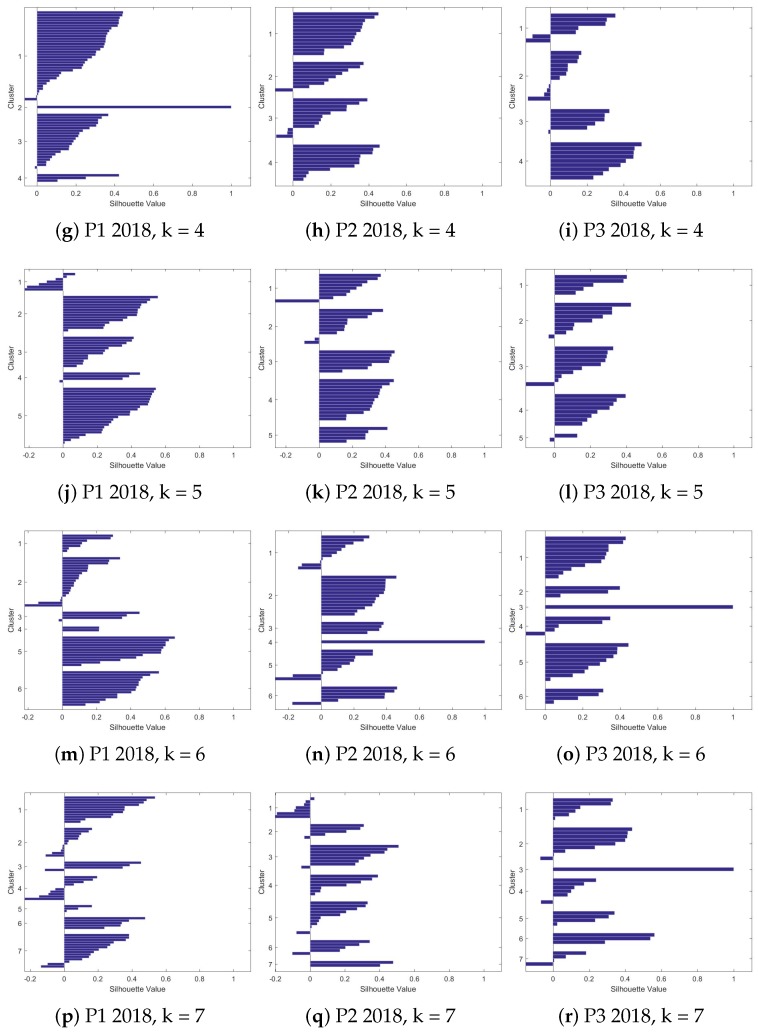
Silhouettes’ charts for three different times in 2018.

We evaluated seven different values of *k* for each of the databases. For each value of *k*, the centroids and the indexes of the databases were saved, in the case of a supervised problem. The evaluation of *Silhouettes* helps to find the best value of *k* for each database, since the number of groups is not known, nor what types of behavior they present. The results of all evaluations for each value of *k* are shown in Figure 5 and Figure 6.

The number of observations in each presentations is different, as explained earlier in Table 2. In this way, the best number of *k* can vary between the databases evaluated. In Figure 6, the first presentation of the second year has, in all evaluated values of *k*, a large group. This can be considered a differentiated behavior in relation to the other two presentations that, as *k* increases, the observations are separating between the clusters.

Observing again Figure 5 and Figure 6, the initial values of *k* have few observations with negative Silhouettes values (indicating that the observation does not belong to the cluster). This suggests that the best number of clusters is in the first values of *k*. For the first year of evaluation (2017), one can also note that the best groupings are in the first two values of *k*. After *k* = 3, the algorithm starts assigning groups with only one observation. There are also many negative Silhouettes values.

As mentioned, it is not always easy to find the right number of groups for an unsupervised learning problem. In this work, the visual technique of Silhouettes was used and the mean values of the Silhouettes were also used in the evaluation. The centroids of each group were also evaluated; for this work, a clear separation between the centroids of the attributes for each group, in a given database, was sought. Table 6 shows the mean values of the Silhouettes. In this stage of the work, the best values of k for each year were found, in order to later be analyzed in each of the databases (presentations) grouped.

### 4.3. Comparison among Clusters - Centroid Analysis

In the presentations of the year 2017, based on both the averages’ table and the visual analysis, it was decided to analyze the values of *k* = 3. It is important to make clear that this value of *k* was also chosen after an initial analysis of the centroids. This was done with the intention of avoiding the high values that the Silhouettes also presented, with little separation between the centroids’ values of the groups. Thus, this high average value might seem like a good value choice for clusters. An example of this behavior can be seen in the value of *k* = 4 for presentation 3, in year 2017, Figure 5i.

For the year 2018, there was a greater variation in the values of *k*. The value of *k* = 3 was chosen again. This value (*k* = 3) presents a good clustering, as can be seen in Figure 6d–f. The average value of the silhouettes does not present such high values, but to compare with presentations for the year 2017, it is important to choose the same value of *k* for the two years.

**Figure 7:**
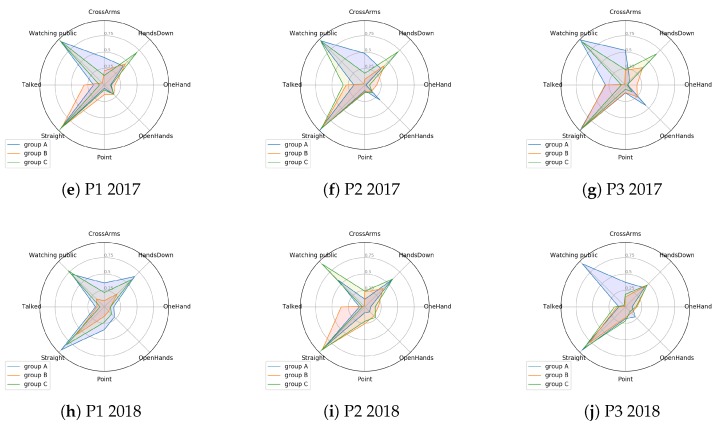
Polar plot of centroids’ values for each presentation.

For this work, the value of *k* = 3 was chosen first because it presents good average values of silhouettes, and also presents a homogeneous formation of clusters, as can be seen in the Silhouettes. Another reason that led to this choice was the fact that there are three distinct patterns, when looking at the centroids. Choosing, for example, *k* = 2, as the number of clusters, would cause one group of students to be inside another cluster. The centroids analysis for *k* = 3 shows that there are subtle but important differences in the learning context that need to be explored. This section will present an analysis of the centroids’ values, between the different presentations at the determined moments. In this step, as previously mentioned, the centroids already generated were evaluated. The objective of this evaluation step is to find something related to the evolution or behavior of each cluster generated based on its attributes. Figure 7 shows some attributes (eight attributes) in a polar graph. We chose not plot the other four attributes given the fact they are not present in the graph since their centroid values were too small.

Some attributes stand out more and others less, in certain clusters formed. The work of [45] presents some characteristics of excellent performances during a presentation, and other characteristics of performances called poor. These characteristics can be related to the attributes evaluated in the present work, thus making clear the behavior of the clusters.

The characteristics that are considered poor are related to withdrawn body language, such as: defensive arm positioning (folded arms, hands in pockets), withdrawn posture and head down. The characteristics considered as excellent presentations by the authors are: open body posture, hand gestures to emphasize points or convey meaning and inclusive eye contact. In the work, other characteristics are pointed out, those that are mentioned here are the ones that more relate with the attributes evaluated in the present work. We can also associate speech with something positive in a presentation, since not talking indicates, in many situations, that the student explained less about the topic.

#### 4.3.1. 2017 Presentations

In the first presentation of 2017, some attributes differ in relation to the clusters. In this analysis, some of these are analyzed, being chosen because of their importance in the behavior of the clusters. The *CrossArms* attribute, for example, presents a significant difference between the three clusters. These types of attributes are highly relevant when the students communicate. This is because, for example, crossed arms are a typical defensiveness gesture [3]. There is a cluster, group A, of observations that is more with this posture, another that is less, group C, and still another group of observations (group B) that is in the middle between the other two. For this presentation, we still have the *Talked* attribute. This presents the same characteristic of the previous attribute, where there are three distinct ‘markings’ in the three clusters: one with a higher value, another as a middle ground and a smaller one.

The watching public attribute behaves differently from the other two. One cluster has a different understanding compared to the other two, which have almost 100% of the presentation time. The same pattern, not for the same clusters, is found in the point attribute. Two clusters (A and C) have low values, and one (group B) has higher values. Most of the other attributes do not clearly separate the clusters. The *OpenHands* variable, for example, is practically the same in all three clusters. Some attributes have little variation, such as *Straight*, this attribute being important as it indicates that the student is not with his head down, which is considered a bad behavior in oral presentations [45]. Another attribute that separates the three clusters is *HandsDown*. Three clear patterns can be seen in Figure 7a.

In the second presentation, the general behavior remained similar to the previous one. For example, we have that the *Watching public* attribute has matched practically the same behavior. In this presentation, it is also possible to identify a group, group C, with the *Talked* attribute with the highest value (32% of presentation total time). However, it may also be noted that the *Talked* attribute has closer values between two clusters than it did in the first presentation. You can also notice a separation in the *CrossArms* attribute, with three very clear groups. In this presentation, the attribute *HandsDown* also appreciated a variation between three behaviors.

Finally, in the third presentation of this year, a new behavior, similar to the previous ones, were observed. On the other hand, the *Talked* attribute presents two groups (A and B) with high values (around 30%); this may indicate that the presenters converged to a more speech behavior over the time (year) that they were presenting. These same two groups still have values close to the *Point* attribute (approximately 10% for both). The *Point* attribute. The attribute *Point* is relevant in oral presentations with the aid of PowerPoints, as pointing can connect the presenter’s ideas [46]. There is also another group that presents low values for these attributes, (*Talked* and *Point*).

#### 4.3.2. 2018 Presentations

The attributes of 2018, for the first presentation, behaved slightly differently than in 2017. First, it can be assumed that all groups have the *Talked* attribute with values less than 10% of the total presentations. The *Point* attribute, like *HandsDown*, has a separation between the three chosen clusters. For the *CrossArms* posture, this separation behavior in the three groups could also be found. In general, this first presentation does not clearly mark three different behaviors. Apparently, the clusters are distributed over the attributes more randomly than in the year 2017.

In 2018, the values of the attributes among the three clusters are distributed in a scale. Group A had higher values, group C values were slightly smaller, and group B had values smaller than the previous two. Thus, one can see from Figure 7d that the first presentation of 2018 had its clusters forming something resembling a scale. This behavior is different from what has been presented so far (the clusters varied according to the centroids within the presentations).

The initial behavior of some randomness of data and behaviors in the first presentation does not occur in the second presentation. When analyzing the data values, it can be observed that the groups are formed with a “rule”, or behavior, much like they were formed in 2017—this behavior being a cluster with a higher value in the attribute, another cluster with an intermediate value, and a third with a lower value than the previous two.

The last presentation of 2018 behaves differently from the rest of that year, as shown in Figure 7f. First, it can be observed from the graph that the centroid values decrease, with respect to the previous presentations. Another point to be observed is that the clusters formed, changing their general rule. For example, we have that the *Watching public* attribute no longer has two clusters with high values and one with low value. In this presentation, two clusters have low values, groups B and C. In general, in 2018, we can not observe the same pattern that happens in 2017; the rule of formation of clusters is not the same, principally, in the last presentation of the year 2018.

#### 4.3.3. Comparison among Years

It is difficult to point out which are the same groups (if any) throughout the year, between presentations. In this sense, the comparison made in the present work will be to identify the way traveled throughout the year, and especially a comparison between the last presentations of each year. With this, we can identify some pattern that was formed during the semester. The results of Table 5 shows that, at the end of each semester, the number of statistically different attributes drops from 6 to 1.

The 2017 year achieved some constancy in patterns. Presentations varied in centroid values; however, the polar chart helped find three behaviors in the three presentations. This behavior can not be seen for the year 2018, where the same behavior (or similarity) between presentations is not evident.

What can be seen in both years (2017 and 2018) is that there is a convergence of some attributes with greater values, in the last presentation. For example, it can be seen that Group A, in two years, has a similar value for the CrossArms variable. In the same way, Group B has attributes with similar values, such as Watching public and CrossArms. Group C presents some variation between the presentations, but it is possible to perceive a similarity between the presentations of the two years.

After all these results, it can be said that three distinct groups were found in the 2017 presentations; this is easy to observe in Figure 7. For 2018, presentations of the same patterns as in 2017 were not observed. For this year, it became more difficult to separate the clusters. They were called Group A, Group B and Group C. This choice was made after an analysis of the Silhouettes and their means. We also took into account the centroid values of these clusters (*k* = 2 returns a better value of silhouette mean), where a clear separation between these three clusters was observed. Larger numbers of clusters (*k* = 5 in the first presentation of 2018) were also possible in specific cases (Table 6). However, for all the other presentations, the average Silhouette value was better at *k* = 3. This can be called a similarity between the years. Similar to the [47], our study was concerned about finding patterns in student interactions in complex learning environments—in this case, presentations. This process can help teachers and educators with an easy and efficient way to quickly analyze students’ body postures.

There are general similarities between the data collected from the years 2017 and 2018. Table 3 shows the similar behavior between the two years for mean values. Figure 4 shows the similarities in attributes evaluated. In particular, at the beginning of the semester, it was noted that the presentations vary. This can be explained by the number of observations found in the first presentation of 2018 (59 observations), a much larger number than in 2017 (40 observations). The nonparametric Wilcoxon rank sum test helps to note this difference. In Table 5, the test (comparison) that had more significant occurrences of attributes was between the first presentations of 2017 and 2018 (six attributes). It can also be seen that, at the end, with the comparison between the last two presentations, the number of these attributes falls to 1. This indicates that there is a greater statistical equality between the students at the end of the presentations.

Another point of the study to be highlighted is the importance of the attribute *Talked*. Due to our experimental setting, this variable can more easily identify certain patterns or even a cluster within all the dataset. Still, the *Talked* can highlight the other characteristics (attributes) of students, since good communicators must also have skills with body gestures [3].

## 5. Conclusions

MMLA can assist in evaluating complex learning environments using data from a variety of sources. In the present study, we found that, during the presentations evaluated by the inferential statistical test, compared between the years 2017 and 2018, the number of attributes decreased from 6 to 1. This indicates that, at the end of the semester, the students presented in a more similar way, between the years. The results also indicate that, for *k* = 3, there are three different groups in the presentations. By 2017, we can see all three behaviors better than in 2018, where the clusters rule does not get very clearly translated across the attributes, for all presentations.

As a general result of the research, we can also note that the visual method of Silhouettes, along with the evaluation of centroids, helps evaluate this type of problem. With these techniques, it was possible to observe the formation of clusters with centroids. Still, with the Silhouettes, it was possible to determine the best value of *k* for the problem, in a general way. It is noted that the Microsoft Kinect sensors were able to create a database that allowed the separation between groups (clusters).

The inferential test among the attributes also showed that the comparisons in the presentations of the same year, presented few attributes with statistical differences. We can take as an example that the comparison between the second presentation of 2018 and the third presentation of 2018 did not result in any statistically different attributes.

There are two main important outcomes of the ability of identifying the student’s body patterns during presentations and analyze their data: one is related to the assessment of the student, and the other is related to the creation of a tool to analyze different groups’ behaviour. The first outcome is that the presentation capture and body pattern detection enables self-assessment from the student. For instance, if a student is talking most of the available time and is not using non-verbal communication efficiently, an automatic report given as feedback may help him to better assess his presentation. In the case of a regular assessment in which the presentation skills are important, along with the professor’s qualitative feedback, he may use the acquired information to give a more quantitative depth to articulate his recommendations to improve the student’s performance.

On the other hand, the ability to analyze statistical data from groups during presentations may be used as a tool to compare students’ profiles across different fields or generations. The information can then be used to define proper education strategies. Going further from our current possibilities, if a model to assess presentations skills is created or adapted by psychologists to use this type of input, this can become a powerful tool to evaluate how internal and external factors can determine the presentation quality or style.

For future work, new analyses should be conducted in different groups of students. These new students would come from different areas of expertise, such as engineering, health sciences and the humanities. Those distinctions could show different behaviors among the areas of knowledge, or it might reveal similar behaviors among them. Allied to this, information visualization techniques could be explored to aid in the detection of patterns obtained from the students coming from the different areas of knowledge. As a way to return immediate feedback to the students, these types of profiles can be further integrated into the software for automatic classification of presentations in real time. It is also expected to test other classification techniques to improve models’ performance.

## Figures and Tables

**Table 1:** Description of each characteristic of the databases.

Features	Description
CrossArms	The presenter crossed both arms.
Downside	The tilt of the presenter is greater than 0.333, with −1 tilted back and 1 tilted forward.
Straight	The tilt of the presenter is between −0.333 and 0.333, with −1 tilted back and 1 tilted forward.
Watching public	The presenter is looking at the audience.
HandOnFace	The presenter has a hand on the chin.
OpenHands	The presenter is explaining with both hands (both hands with arms folded).
HandsDown	The presenter is holding hands down.
OneHand	The presenter is explaining with one hand down and the other doubled in an explanatory position.
HandOnHip	The presenter has his hands on his waist.
HandOnHead	The presenter has a hand at the nape of the neck.
Point	The presenter is pointing with one hand.
Talked	Presenter voice is detected.

**Table 2:** Number of observations of each database.

Year	Presentation	Number of Students
	P1	40
2017	P2	22
	P3	22
	P1	59
2018	P2	45
	P3	34

**Table 3:** Average values of each attribute.

	2017			2018		
	P1	P2	P3	P1	P2	P3
CrossArms	0.249	0.247	0.281	0.207	0.188	0.243
Downside	0	0.005	0	0.012	0	0
Straight	0.930	0.935	0.939	0.808	0.901	0.856
WatchingPublic	0.493	0.529	0.496	0.473	0.468	0.365
HandOnFace	0.074	0.061	0.053	0.112	0.072	0.084
OpenHands	0.201	0.209	0.249	0.148	0.170	0.157
HandsDown	0.506	0.447	0.427	0.465	0.518	0.442
OneHand	0.132	0.102	0.100	0.111	0.148	0.145
HandOnHip	0.034	0.048	0.026	0.050	0.067	0.041
Point	0.105	0.099	0.099	0.230	0.183	0.180
HandOnHead	0.001	0.003	0.002	0.009	0.003	0.004
Talked	0.212	0.249	0.222	0.107	0.169	0.124

**Table 4:** The top 5 attributes for each database.

		2017			2018	
	P1	P2	P3	P1	P2	P3
1	Straight	Straight	Straight	Straight	Straight	Straight
2	HandsDown	Watching public	Watching public	HandsDown	Watching public	HandsDown
3	Watching public	HandsDown	HandsDown	Watching public	HandsDown	Watching public
4	CrossArms	Talked	CrossArms	Cross Arms	Cross Arms	Point
5	Talked	CrossArms	OpenHands	Point	Point	CrossArms

**Table 5:** Inferential tests between all databases evaluated.

	2017			2018			2017X2018		
	P1xP2	P1xP3	P2xP3	P1xP2	P1xP3	P2xP3	P1xP1	P2xP2	P3xP3
CrossArms	0.900	0.622	0.741	0.338	0.590	0.310	0.309	0.197	0.459
Downside	**0.046**	0.290	**0.017**	**0**	**0.001**	0.744	**0**	**0.023**	0.251
Straight	0.213	0.926	0.369	**0.006**	0.084	0.425	**0.003**	0.607	0.510
Talked	0.361	0.954	0.495	0.060	0.493	0.282	**0**	**0.030**	0.065
Watching public	0.888	0.684	0.829	0.624	0.065	0.590	0.917	0.376	0.168
HandOnFace	0.842	0.357	0.357	0.669	0.804	0.831	0.509	0.952	0.317
OpenHands	0.653	0.082	0.346	0.321	0.734	0.666	**0.025**	0.179	**0.007**
HandsDown	0.327	0.216	0.657	0.259	0.876	0.164	0.283	0.248	0.763
OneHand	0.168	0.497	0.593	**0.029**	0.102	0.909	0.493	**0.017**	0.102
HandOnHip	0.906	0.054	0.108	0.952	0.105	0.150	0.723	0.784	0.604
Point	0.900	0.858	0.691	0.309	0.352	0.964	**0.002**	0.062	0.086
HandOnHead	0.264	0.138	**0.029**	0.291	0.050	0.384	**0.030**	0.826	0.189
Total	1	0	2	3	1	**0**	6	3	1

**Table 6:** Silhouettes’ average values.

		2017			2018	
	P1	P2	P3	P1	P2	P3
*k* = 2	0.417259	0.478511	0.437844	0.280178	0.38786	0.380755
*k* = 3	0.310087	0.382019	0.44329	0.259083	0.2838	0.36101
*k* = 4	0.227186	0.192535	0.310865	0.237299	0.247055	0.190905
*k* = 5	0.173825	0.286057	0.247999	0.274679	0.255968	0.204827
*k* = 6	0.19337	0.232454	0.20892	0.26236	0.23061	0.268057
*k* = 7	0.217924	0.303317	0.253029	0.165628	0.163419	0.21593
